# The Pearl upon the Rose*Dr. Edward Jenner’s figurative description of the vaccine vesicle. See Barnou’s Life of Jenner, Vol. ii, p. 303.

**Published:** 1888-10

**Authors:** Henry A. Du Bois

**Affiliations:** San Rafael, Cal.


### "THE PEARL UPON THE ROSE."*

BY HENRY A. DU BOIS, PH. B., M.D., SAN RAFAEL, CAL.

An examination of the latest systematic work on the practice of medicine—that of the late Dr. Fogge—fails to discover any description of vaccination, much less any account of the vaccine vesicle and its origin and mode of development; and after a somewhat extensive search through the text-books one finds himself compelled to go back to the original works of Jenner published in 1798, and to the very able monograph of Robert Ceely published as late as 1840, for an accurate description of the vaccine vesicle and its normal and abnormal development, clearly illustrated by colored plates. Our opinions of disease are at present in a transition stage. Darwin's generalizations have been absorbed and become a part of the thoughts of all students of science. The germ theory, with or without its appendix of the animal alka-

*Dr. Edward Jenner's figurative description of the vaccine vesicle. See Barnou's Life of Jenner, Vol. ii, p. 303.

loids, has quickly followed. These wide generalizations will find their strongest proof or receive a decisive defeat from comparative medicine, a science hardly yet born, but which (in my opinion at least) has a most brilliant career before it. By comparative medicine I mean a complete history of the origin and development of diseases in all organisms, vegetable as well as animal. This involves the tracing of diseases already studied in man, through other animals, and even it may be into vegetable organizations, and from the facts thus collected by thousands of observers, deducing the laws governing diseases. Such observations will doubtless show that diseased action varies according to the organization in which it exists; so that the symptoms presented to the observer may vary greatly according to the animal in which they are studied. As we in our bodies retain traces (apparently useless) of the plan on which the whole class of vertebrates are constructed, so, doubtless, with our diseases, a connection will in time be established with those of other animals. *Nothing, it seems to me, is more logical than the conclusion that, as our bodies are formed on the same general plan as that of other vertebrates, so our diseases are of necessity closely allied, and if variations take place in the structure of varieties or species, so may variations in the disseases follow change of structure.* In other words, if an animal designated as A can change in structure so as to resemble an animal designated as B, or become identical with it, it logically follows that the diseases of A will resemble or be identical with B. In germ diseases the germs doubtless are subject to the laws governing vegetative growth. A high temperature accelerates their development, and the readiness with which they are able to assimilate the elements necessary for their growth, probably has no inconsiderable influence in the causation of the symptoms presented. The orientals introduced the germs of a disease into the skin, and thus caused their absorption by the lymphatics and the consequent modification of the diseases from that caused by the same germs entering the system bv the lungs. Jenner in vaccination went a step further by modifying the germs before inoculation, by their passage through the economy of an animal with a higher temperature than man. That Jenner believed in a modified small-pox, the name which he uniformly used, shows conclusively. He always wrote and spoke of the cow small-pox—"the variolæ vaccinæ"—not of the cow-pox. He certainly believed in a cow-pox only as a modification of small-pox, or, as modern science would now say, an attenuation of its virus. Robert Ceely inoculated a cow with small-pox and with the resulting virus he inoculated children, producing typical vaccine vesicles, from which over two thousand persons were successfully vaccinated within a few months. He went a step further and inoculated a cow first with small-pox, and then after the lapse of some days he vaccinated the same animal, with the result of producing two sets of vesicles precisely alike, except that those resulting from the small-pox virus were more pronounced and appeared later than those resulting from the vaccine virus * Mr. Badcock, of Brighton, England, out of some five

*Observations on the Variolæ Vaccinæ, by Robt. Ceely, p. 381, published in Trans. of the Provincial Medical and Surgical Association, Vol. viii and x, Worcester England, 1840. See plates 9 to 14 inclusive.

hundred or six hundred inoculations with small-pox virus, succeeded in producing well formed vesicles in thirty-seven cows, or seven per cent. of those operated on, so difficult did he find successful inoculation to be; and from the virus thus obtained thirty thousand persons were vaccinated by him, and the virus is still in use in England.† All this was done nearly half a century ago, and yet we read in an article by a practical vaccine propagator in a recent system of medicine* "that small-pox and cow-pox are wholly distinct from each other under all circumstances, and that it is impossible to convert the one into the other"; again, "this practice" (inoculating cows to obtain vaccine virus) "is utterly fallacious, and it is also dangerous, since the disease as produced, however mild it may seem to be, is nothing more nor less than small-pox, with its infectiousness by effluvium and its liability to prove serious, even when carefully inoculated"; and again, "a method that in the light of our present knowledge can only be characterized as downright malpractice." This all on the authority of Chauveau of the Lyons Commission, and against the opinion of Jenner, the opinion and practice of Ceely and Badcock, and the almost universal opinion held by English physicians from the time of Jenner's discovery until the present day, as well as by a multitude of Continental and American experimenters. Barron (Report on the Present State of Vaccination, Vol. viii, p. 20, Trans. Prov. Med. and Surg. Soc., 1840) claims that cow small-pox appears in cattle in two forms, one epidemic of only occasional occurrence, very fatal and infectious; the other milder and non-infectious. The former frequently has occurred, he says, at the same time as an epidemic of small-pox in man, and in India is designated by the same names applied to variola; viz., "bussunt, whatta or gotee."†

Let us now see what the Lyons Commission have proved:

1. They claim to have inoculated cows with small-pox; did they really do so?

2. That the cows so inoculated were proof against cow-pox; were they? And lastly, that "the small-pox in its passage through the system of the cow is not transformed into vaccinia; it remains small-pox and returns to the original state of small-pox when introduced into the human species."* Altogether, the commission claims to have inoculated seventeen cows and three horses without a failure. Badcock only, as we have already seen, succeeded in thirty-seven inoculations out of between five hundred and six hundred, or about seven per cent. Ceely met similar difficulties,

---

†A detail of experiments confirming the power of cow-pox, etc., as cited by Seaton in his Handbook of Vaccination, Eng. Ed., 1868.

*Vaccinia, by Frank P. Forter, M. D., being article in Pepper's Prac. Med. 1885; see p. 457.

†An Inquiry into the Causes and Effects of the Variolæ Vaccinæ, etc., by Edward Jenner, from second London edition, reprinted at Springfield (with plates) 1802. Further Observations on the Variolæ Vaccinæ, etc., bound with the above. These plates illustrate well the vesicle, but cannot be compared with the artistically colored plates in the monograph of Ceely. These present, as does the whole work, evidences of the most minute accuracy, are carefully colored by hand, and number nearly, if not quite, one hundred separate illustrations. For this work I am indebted to the kindness of Dr. Billings of the Surveyor General's Library, who kindly put me in the way of obtaining a copy of this rare and very valuable original monograph.

and such seems to have been the experience of experimenters generally. They claim that in ten of these, vaccination took more or less in four, and failed in six. On vaccinating three children with virus obtained by scraping the papules after their removal from the cow, small-pox was produced. In weighing conflicting evidence it is important to know the character of the men giving it. In this case we have Robert Ceely's papers with accompanying plates to judge him by, which unfortunately the Lyons commission do not seem to have consulted. Both reports and plates show excessive care and the most minute accuracy. The statements made in the report of M. Chauveau are in oppisition in many respects to the facts as given by experimenters generally, and many of them seem to be made carelessly and recorded from memory some time after the experiments had been made. Briefly they state that the inoculation of small-pox in the cow produces a local eruption of pimples "so minute as to escape notice unless one is on the look-out for them"; and which they failed themselves to recognize in the first five cows experimented on, and they say "that most persons who saw this eruption considered that it was the result of inflammatory action round the inoculated part, and was not indicative of a specific infection."

Robert Ceely before, and LeBlanc and Depaul afterward, obtained the same eruption, and neither of them considered that it indicated an infection of the system. Ceely obtained in his successful cases, not an eruption of minute pimples with scanty or no secretion, but large vesicles with all of the characteristics that go to make up the typical vaccine vesicle. Badcock and Depaul, so far as I can ascertain, obtained a similar eruption. How are we to account then for cow-pox failing to take in six out of the ten cows if the eruption was not that of small-pox? From an experience in the vaccination of over one hundred cows and calves I can safely say that it is no unusul thing for several cows vaccinated with great care and fresh virus (successful in other animals) to fail to take, to develop no vesicles or developing vesicles, to furnish "barren virus." Ceely would probably say that they had failed from some of the many causes given in his papers (page 399) in producing cow-pox. Probably the most likely reason for the non-action of cow-pox in the six cows is that it followed the previous inoculation too soon; so that the skin was in an irritated condition that did not favor absorption of the virus. Lastly, how are we to explain the fact of the three children taking small-pox after being vaccinated with the virus said to have been thus produced? Small-pox seems to have prevailed at Lyons at the time these experiments were being made, and we are told nothing of their surroundings. In 1799 Woodville found when he vaccinated with cow-pox direct from the cow, but with persons surrounded by the effluvium of small-pox in a hospital, that three-fifths of his cases developed a general eruption which proved to be small-pox. Jenner convinced him that small-pox had gained access to the system but not through the virus used, as that when used elsewhere produced typical pocks. Again, if the eruption in the cow was only the result of irritation, as they say many competent observers thought, it is possible that the small-pox virus used may have been simply transferred from the skin of the cow, where it had remained unabsorbed, to that of the child, so that when used on the child it exerted its full force and produced confluent small-pox which resulted fatally in one case at least. To sum up briefly, Ceely's experiments are exact, carefully made and recorded. and above all, his plates enable us to judge of the vesicles that he obtained. From these I think one must conclude that after inoculating with small-pox virus, he obtained vesicles having all the appearances of cow-pox vesicles, and that on the same animal, by a vaccination of a few days later than the inoculation, he succeeded in obtaining two vesicles, one the result of small-pox virus and the other the result of cow-pox irus, and both alike.

The report of the Lyons Commission as we have already said is not minute enough, and there are no carefully colored plates to enable us to judge accurately of the vesicles obtained. There seems evidences of haste. The eruption produced was wholly unlike that considered typical by other experimenters. They were uniformly successful in their inoculations, while all others have failed in at least nine out of ten animals experimented upon. The greatest care and cleanliness is required in experiments of this kind where two viruses are in use at the same time, and may become mixed, the one with the other. The weight of evidence, it seems to me, is in favor of the modified identity of these two diseases. In 1864M. Depaul said: "There is no vaccine virus. Vaccine is nothing but modified small-pox, attenuated in its passage through the system of the horse or cow."

Warlomont, of Brussels, indeed has recently changed his belief in this matter, and whereas he formerly believed the two diseases distinct, he now believes both diseases are caused together with equine, by one virus attenuated in different degrees by the temperature and other surroundings that it meets in each animal, and here for the present we leave the vaccine vesicle.†—*Cal. Prac.*

*See Report of commission as quoted by Trousseau in his Clinical Medicine. Vol. ii, p. 118. Also Vaccine e Variole per M. M. Chauveau Vicunois et Maynet, in memories et Comptes Rendus de la Soc. des Sci. Med. en Lyon, Vol. v.

*† A Manual of Animal Vaccination, Dr. E. Warlomont, translated by A. G. Harries, M.D., 1886. Philadelphia.

